# Strontium and gallium doping enhances *in vivo* bone regeneration in biomimetic hydroxyapatite 3D-printed scaffolds

**DOI:** 10.1016/j.mtbio.2026.103131

**Published:** 2026-04-16

**Authors:** Irene Lodoso-Torrecilla, Daniel Moreno, Gaël Ciucci, Miguel Mateu-Sanz, Ji-Young Yoon, Emilio Jimenez-Pique, Jordi Franch, Maria-Cristina Manzanares, Joanna Konka, Montserrat Espanol, Maria-Pau Ginebra

**Affiliations:** a Department of Materials Science and Engineering, Group of Biomaterials, Biomechanics and Tissue Engineering, Universitat Politècnica de Catalunya (UPC), Barcelona, Spain; b Barcelona Research Centre for Multiscale Science and Engineering, Universitat Politècnica de Catalunya (UPC), Barcelona, Spain; c Centro de Investigación Biomédica en Red—Bioingeniería, Biomateriales y Nanomedicina (CIBER-BBN), Instituto de Salud Carlos III, Spain; d Department of Materials Science and Engineering, CIEFMA Group, Universitat Politècnica de Catalunya (UPC), Barcelona, Spain; e Bone Healing Group, Small Animal Surgery Department, Veterinary School, Universitat Autonoma de Barcelona, Bellaterra, Barcelona, 08193, Spain; f Human Anatomy and Embryology Unit, Department of Pathology and Experimental Therapeutics, Universitat de Barcelona, L'Hospitalet de Llobregat, Barcelona, 08907, Spain; g Institute for Bioengineering of Catalonia (IBEC), Barcelona Institute of Science and Technology, Carrer Baldiri Reixac 10- 12, Barcelona, 08028, Spain

**Keywords:** 3D printing, Calcium phosphates, Biomimetic hydroxyapatite, Osteoinduction, Ion doping, Scaffold, Bone regeneration

## Abstract

Doping of calcium phosphates (CaPs) with bioinorganic ions is a widely used strategy to enhance their biological performance in bone regeneration. However, conventional methods for ionic incorporation in CaP scaffolds often require high-temperature treatments or involve multiple complex steps. Here, we present two simple strategies to dope 3D-printed CaP scaffolds via incorporation of ions into the apatitic phase during the hydrolysis of α-tricalcium phosphate (α-TCP) to calcium deficient hydroxyapatite (CDHA). In the first strategy, ions were incorporated directly into the printing ink, whereas in the second, undoped robocasted scaffolds were immersed in ionic solutions, allowing ion incorporation into precipitated CDHA during phase transformation. We investigated several ions, including strontium (Sr^2+^), magnesium (Mg^2+^), silicon (SiO_4_^4−^) and gallium (Ga^3+^). Sr^2+^ and Ga^3+^ were successfully incorporated into the scaffolds, either by direct ink doping (Sr^2+^) or by soaking in ionic solutions (Sr^2+^ and Ga^3+^). Direct incorporation of Sr^2+^ in the ink resulted in a higher ion loading and release, enhancing bone formation and bone quality, as evidenced by increased mineral-to-matrix ratio and Young's modulus, as well as osteoinductive properties relative to non-doped scaffolds. Furthermore, we demonstrated for the first time the osteoinductive capacity of Ga^3+^ in an ectopic *in vivo* model.

## Highlights

• Simple strategies enable ion doping of 3D-printed calcium phosphate scaffolds.
• Sr^2+^ and Ga^3+^ successfully incorporated via ink or post-soaking methods.
• Direct Sr^2+^ ink doping enhances ion release, bone formation, and scaffold stiffness.
• Ga^3+^ shows osteoinductive potential in an ectopic *in vivo* model.

## Graphical abstract

**Figure:**
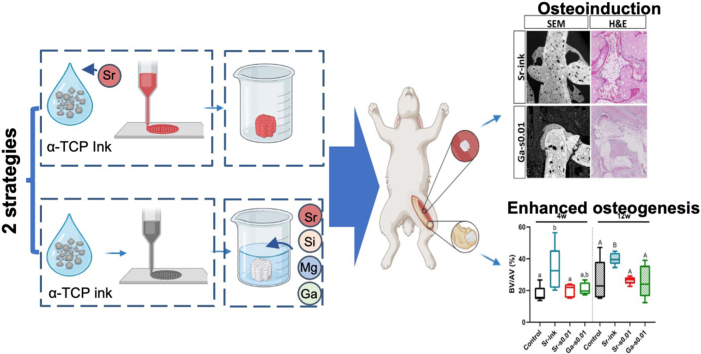

## 1 Introduction

The increasing prevalence of bone diseases linked to the aging population calls for the development of advanced biomaterials and improved synthetic bone grafts. Among the various options, calcium phosphates (CaPs) are widely used due to their close resemblance to the inorganic phase of bone as well as their biocompatibility and bioactivity [1,2]. In particular, calcium-deficient hydroxyapatite (CDHA), which can be synthesized by hydrolysis of α-tricalcium phosphate (α-TCP) at physiological temperature, stands out as a particularly promising candidate. In addition to its excellent *in vivo* response [3,4], CDHA exhibits self-hardening properties that provide significant processing versatility, enabling its use in applications ranging from calcium phosphate cements and foams [5] to self-hardening inks for 3D printing [6,7]. The hardening process involves the hydrolysis of α-TCP, resulting in an entangled network of nanometric CDHA crystals.

In recent years, ion doping of CaPs has attracted considerable attention as a promising strategy to enhance their biological performance [[8], [9], [10], [11]]. The incorporation of ions can improve the bioactivity of these biomaterials through two complementary mechanisms. First, certain ions can act directly as therapeutic agents, an approach often referred to as ionic medicine, by being released at the target site to support bone remodeling and treat bone diseases. Second, ion incorporation can indirectly enhance bioactivity by modifying the textural, surface or solubility properties of a material.

Similarly to natural bones, synthetic hydroxyapatite (HA) can allocate ionic substitutions [[12], [13], [14]]. HA can be doped by different chemical routes, the aqueous precipitation method at low temperatures being one of the most common methods. Depending on the type of ion, the mechanism of incorporation in nanostructured apatites, either substituting another ion in the crystal lattice or being incorporated in the so-called hydrated layer on the crystal surface, influences the maximum doping concentration [15,16].

The most widely used ions are strontium [8,17,18], magnesium [9,19,20], silica [10,21,22] or zinc [[23], [24], [25]], elements naturally present in the inorganic phase of bone. Strontium (Sr^2+^) has total solubility in the apatitic structure and can progressively replace Ca^2+^ ions in the crystal lattice up to 100% substitution, inducing a linear expansion of the lattice constants [26,27]. It has been shown to have a positive effect on bone remodeling by increasing osteoblast activity and inhibiting osteoclast-mediated resorption [[28], [29], [30]]. Magnesium (Mg^2+^) plays an essential role in bone metabolism by enhancing cell proliferation and differentiation and angiogenesis and it has been shown to prevent risk factors associated with osteoporosis [31,32]. Unlike Sr, the incorporation of Mg ions into HA is limited, around 10 at.% (≥2.5 wt%) [33,34]. Moreover, it has been reported that it is mainly incorporated in the metastable hydrated layer on the surface of the apatitic nanocrystals [35]. Silicon is a key element in bone formation and growth and it has been demonstrated to enhance the bioactivity and osteogenic potential of the scaffolds [[36], [37], [38]]. Si is incorporated into the HA structure in the form of silicate ions, (SiO_4_^4−^) by replacing phosphate ions, (PO_4_^3−^), which requires charge compensation mechanisms in the form of HPO_4_^2−^ ion formation, cationic co-substitutions or OH^−^ vacancies [[39], [40], [41]]. The highest Si substitution value for HA reported in the literature is 5 wt% [41]. Zinc ions (Zn^2+^) are capable of stimulating osteoblast cell proliferation and differentiation, and they also exhibit osteogenic effects [42]. In a recent systematic review, the effect of the doping of these ions into CaP-based bone substitutes was analyzed and it was observed that CaPs with strontium, magnesium and silica exhibited a higher bone regeneration than their ion-free counterparts. Zinc, on the other hand, did not show any beneficial effect on bone regeneration [11].

Most recently, other ions with antibacterial properties, such as copper, silver or gallium, have been incorporated into CaPs. However, optimizing the concentration of copper or silver is challenging due to their cytotoxicity at high concentrations. Gallium (Ga^3+^), in contrast, has been recently proposed as a therapeutic ion. Owing to its chemical similarity to Fe^3+^, Ga^3+^ can disrupt various metabolic processes in both cells and bacteria [[43], [44], [45]]. Beyond its antibacterial effects, Ga^3+^ has been shown to enhance bioactivity and mineralization in osteoblast-like cells [46], inhibit osteoclastogenesis and promote differentiation of human mesenchymal stem cells (hMSCs) into osteoblasts in a dose-dependent manner, thereby facilitating osteoinduction [47]. Despite these promising properties, the incorporation of gallium into CaPs remains largely unexplored, and its osteoinduction potential has not yet been demonstrated in a relevant *in vivo* model.

In this work, we investigate low temperature routes for ionic doping of 3D-printed biomimetic hydroxyapatite scaffolds as an alternative to conventional high-temperature ceramic methods. The conceptual innovation lies in the integration of biomimetic synthesis with 3D-printing technology, overcoming the limitations of conventional high-temperature ceramic processing. Traditional ion-doped apatites typically require sintering at temperatures exceeding 1000 °C, which leads to high crystallinity, excessive grain growth and reduced surface reactivity. In contrast, our low-temperature ionic doping strategies are conducted under physiological conditions (37 °C in an aqueous environment), mimicking the natural biomineralization process. This results in a doped apatite phase characterized by high specific surface area, low crystallinity, and structural properties remarkably similar to biological apatite, with enhanced bioactivity and osteogenic properties [3,4]. Crucially, the low-temperature approaches proposed are fully compatible with microextrusion 3D-printing, enabling the fabrication of patient-specific, complex-geometry implants.

Our focus is on scaffolds produced from self-hardening inks. Similar to calcium phosphate cements, ion doping can be achieved during the self-hardening reaction, which involves a dissolution/precipitation process and typically entails adding the desired ion to either the liquid or powder phase [48,49]. However, modifying the formulation of self-hardening, printable pastes for 3D printing can significantly affect their rheological and hardening properties, necessitating extensive ink optimization. In this study, we examine the feasibility of doping CDHA scaffolds with Sr^2+^ by incorporating a strontium salt into the powder phase of a printable α-TCP paste.

Alternatively, we propose a more straightforward approach for doping CDHA with different ions based on the ability of the 3D-printed scaffolds to harden while immersed in a soaking solution. We hypothesize that performing the α-TCP hydrolysis of as-printed scaffolds in ionic solutions as opposed to distilled water may result in the formation of ion-doped CDHA as the final product. A key benefit of this approach is that it eliminates the need for laborious and time-consuming ink optimization each time the formulation is modified to incorporate different ions. As a result, it becomes feasible to incorporate the desired ion(s) during the curing process using a single starting ink formulation. Here, we explore the possibility of incorporating Sr^2+^, and also other ions such as Mg^2+^, SiO_4_^4−^ and Ga^3+^ in the structure of 3D-printed CDHA scaffolds using this approach. After thoroughly analyzing the effects of ionic doping on the physicochemical and mechanical properties of the 3D-printed scaffolds, we evaluate the biological performance of the most promising groups in a rabbit model, focusing on both osteoconductive and osteoinductive properties.

## 2 Materials and methods

### 2.1 Scaffold preparation

The inks, consisting of ceramic suspensions were obtained by mixing a 30 wt% poloxamer 407 (Kolliphor® P 407, BASF pharmaceuticals, Germany) aqueous solution with α-tricalcium phosphate (α-TCP) powder in a liquid-to-powder ratio (LPR) of 0.5 g/g. The mixing was conducted at room temperature in a dual asymmetric centrifugal mixer (DAC 150; Speedmixer, USA). α-TCP was prepared as described previously [50]. Shortly, a mixture of CaHPO_4_ (Sigma Aldrich, USA) and CaCO_3_ (Sigma Aldrich, USA) with a 2:1 M ratio was sintered at 1400 °C for 2 h and rapidly quenched to room temperature. The resulting material was ball milled to obtain fine α-TCP powder and subsequently sieved using a 40 μm sieve (Filtra, Spain). The final α-TCP particles had a median particle size of 3.12 μm and a mean particle size of 5.55 μm.

The resulting self-setting inks were printed using a custom-made DIW printer (Fundació CIM, Barcelona, Spain). The scaffolds were printed using a nozzle with an orifice diameter of 250 μm (25 ga. tapered tip, Nordson EFD, USA) with an orthogonal pattern, 50 % infill and 0.2 mm layer height. For scaffold characterization, cylindrical scaffolds with 6 mm in diameter and height were printed. Instead, for *in vivo* characterization scaffolds with 5 mm in diameter and 8 mm height were printed.

### 2.2 Ion incorporation

**Fig. 1:**
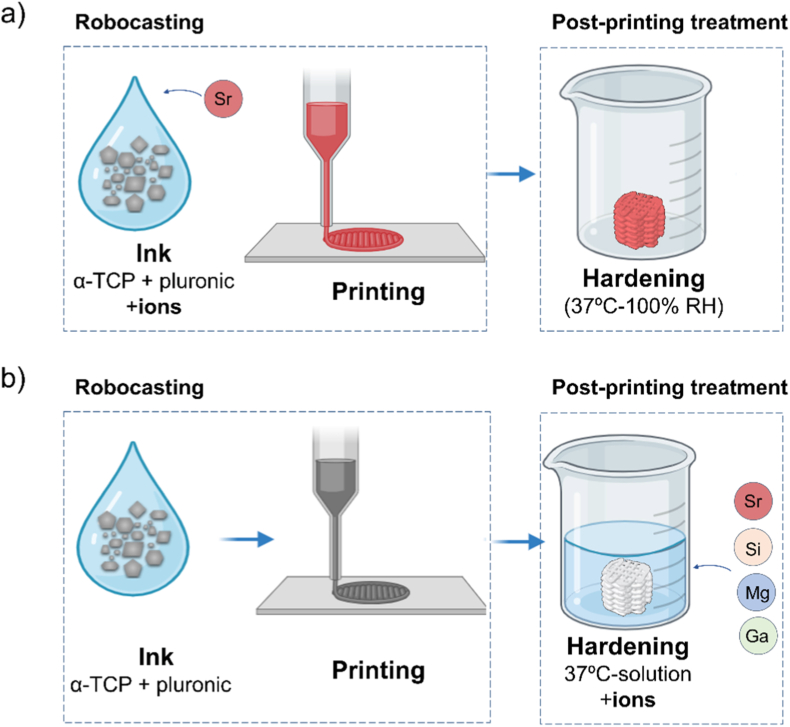
Routes followed for doping the robocasted CDHA scaffolds: a) addition of ions directly in the ink and b) addition of ions during the post-printing treatment, by soaking the scaffolds in an ion-containing aqueous solution. The hardening time was 7 days in both cases.

Two different routes were followed for doping the robocasted CDHA scaffolds, as displayed in Fig. 1: i) the addition of ions directly in the ink, before printing (Fig. 1a); and ii) the addition of ions to the soaking solution where the hydrolysis and consequent hardening take place, during the post-printing treatment (Fig. 1b).

In the first approach (Fig. 1a), Sr was incorporated into the ink by mixing strontium chloride hexahydrate in powder form (SrCl_2_ × 6H_2_O, Sigma Aldrich, MO, USA) with α-TCP in a 20 wt% and following the procedure described above for ink preparation. After printing, the scaffolds were incubated for 7 days at 100 % relative humidity and 37 °C to allow for the hydrolysis to take place but simultaneously avoiding ion release during this process. This group was named Sr-ink.

In the second approach (Fig. 1b), the ions were incorporated in the scaffolds post-printing via an immersion-based doping process. Briefly, a printing paste was prepared by mixing α-TCP powder with a 30 wt% poloxamer 407 aqueous solution. Following 3D printing, the scaffolds were incubated at 37 °C and 100 % relative humidity (RH) for 3 h to enhance structural cohesion. Subsequently, the scaffolds were immersed in aqueous ionic solutions for 7 days at 37 °C to facilitate the simultaneous hydrolysis of α-TCP into CDHA and the concomitant incorporation of the dopant ions. Ionic soaking solutions (0.01 and 0.1M) containing strontium, magnesium, silicon or gallium were prepared by dissolving the following salts in deionized water: strontium chloride hexahydrate (SrCl_2_ × 6H_2_O, Sigma Aldrich, MO, USA), magnesium chloride hexahydrate (MgCl × 6H_2_O, Panreac, Germany), tetraethyl orthosilicate (TEOS) (Si(OC_2_H_5_)_4_, Sigma Aldrich), and gallium nitrate (Ga(NO_3_)_3_, Sigma Aldrich), respectively. After incubation for 7 days in the ionic solutions, the scaffolds were rinsed three times (5 min each) to remove residual salts. The resulting groups were designated by the chemical symbol of the dopant, followed by 's' (soaking) and the molar concentration of the soaking solution; for example, Sr-s0.01 denotes scaffolds immersed in a 0.01 M strontium chloride solution.

### 2.3 Chemical characterization of the scaffolds

For the initial assessment of all studied groups and the selection of the most relevant groups for further analysis, the phase composition of the scaffolds was analyzed by X-ray diffraction (XRD), and the amount of incorporated ion was measured by Inductively Coupled Plasma (ICP) and Energy-dispersive X-ray spectroscopy (EDS). XRD was performed using a diffractometer (D8 Advance Eco, Bruker AXS GmbH, Germany) equipped with a Cu Kα X-ray tube (λ = 0.1541 nm), a Ni filter and a LynxEye detector. Measurements were acquired in the 20-60° 2θ range with a step size of 0.02° and a sampling step time of 2 s. Prior to the measurements, the samples were manually crushed using an agate mortar. The quantification of the crystalline phases was performed by Rietveld refinement using Profex 5.0.1 [51]. ICP-Optical Emission Spectrometry (ICP-OES, 5100 SVDV, Agilent Technologies, Japan) was used to quantify the amount of incorporated ion as well as the amount of Ca^2+^ and P_i_. As the gallium incorporation was expected to be low, ICP-Mass Spectrometry (ICP-MS, 7800 ICP-MS, Agilent Technologies) was used. Prior to measurement, the samples were ground and digested in HNO_3_ (PanReac AppliChem, ITW Reagents). Moreover, the elemental composition of the surfaces and cross sections of the scaffold strands was determined by energy-dispersive X-ray spectroscopy (EDX) with an ULTIM MAX 100 detector (OXFORD Instruments, UK), in a field emission scanning electron microscope TESCAN AMBER.

### 2.4 Ion release

To determine the ion release, a 3D-printed scaffold was placed in 1.5 mL of Tris(hydroxymethyl)aminomethane (TRIS) 0.1 M solution adjusted at physiological pH. The supernatant was changed at each time point (t = 5 h, 1, 2, 5, 7 and 14 days). The release of Ca^2+^ and the doping ion from the scaffolds was determined through ICP-MS. The release tests were done in triplicate.

### 2.5 Microstructure and porosity characterization

The microstructure of the scaffolds was analyzed through scanning electron microscopy (SEM, Neon 40, Zeiss, Germany) operating at 15 kV. Before SEM analysis, the surface of the samples was coated with a carbon evaporated layer (K950X Turbo Evaporator, Emitech, France) to prevent electrostatic charges. The specific surface area (SSA) was determined by nitrogen adsorption using the Brunauer-Emmett-Teller (BET) method (ASAP 2020, Micromeritics, GA, USA), and the pore entrance size distribution and intrastrand porosity were determined by mercury intrusion porosimetry (MIP) [52].

### 2.6 Mechanical characterization

A uniaxial compression test in the Z printing axe was carried out in a universal testing machine (BIONIX, MTS Systems Corporation, USA) with 2.5 kN load cell. The displacement rate was 1 mm min^−1^. For each sample group, 12 scaffolds were tested, which were previously wetted overnight to mimic the *in vivo* situation.

### 2.7 Cytotoxicity assessment

To evaluate the biological safety of the low-temperature doped scaffolds, *in vitro* cytocompatibility was assessed using extracts from 3D scaffolds in accordance with ISO 10993-5 and ISO 10993-12. Briefly, 3D scaffolds were sterilized with 70% ethanol (EtOH) for 30 min and washed three times with phosphate-buffered saline (PBS). Extracts were prepared by immersing the 3D scaffolds in growth medium at a ratio of 0.1 g/mL and incubating at 37 °C for 72 h. After incubation, the extracts were collected and diluted to final concentrations of 100%, 50%, and 25% using fresh growth medium. Saos-2 (ATCC) cells were cultured in growth medium (GM) consisting of Dulbecco's Modified Eagle Medium (DMEM) supplemented with 10% fetal bovine serum (FBS), 1 % penicillin/streptomycin (P/S), and 1% GlutaMAX (Gibco). One day prior to extract treatment, Saos-2 cells were seeded at a density of 1 × 104 cells per well in a 96-well plate. Cells were exposed to the extracts for 24 h, after which cell viability was assessed using a CCK-8 assay kit (Dojindo, Japan). The OD values were measured at 450 nm and the background absorbance at 650 nm was subtracted using a multimodal plate reader (Tecan). Cell viability was normalized to the corresponding medium-only group consisting of growth medium incubated under identical conditions and dilution ratios but without material, and results were expressed as a percentage.

### *2.8* *In vivo* characterization

#### 2.8.1 Study design

Animal procedures were performed in compliance with the Guide for Care and Use of Laboratory Animals [53] and the European Community Guidelines (Directive 2010/63/EU) for the protection of animals used for scientific purposes and under the permission of the National Animal Ethics Committee on Human and Animal Experimentation (Approval # CEAAH 4683). The study was performed on New Zealand White rabbits with a body weight ranging from 3.7 to 5.7 Kg and an age range of 8-12 months. The animals were purchased from a professional stock breeder and housed in individual 2 m^2^ boxes during the study. Before surgery, a two-week acclimatization period was established.

Cylindrical scaffolds (5 mm in diameter and 8 mm in height) were printed and, after hardening, sterilized at 25 kGy. Four experimental groups were analyzed: control, Sr-s0.01, Ga-s0.01 and Sr-ink, with two time points, 4 and 12 weeks. Bilateral femoral condyle defects were used to analyze osteoconduction, and bilateral intramuscular pockets were used to assess osteoinduction in the 12-week group, with n = 6 for each material and time point.

#### 2.8.2 Surgical procedure

For the surgical procedure (Fig. S1), the animals were pre-anesthetized using butorphanol (0.5 mg/kg, s.c.), midazolam (0.5 mg/kg, s.c.), and medetomidine (0.05 mg/kg, s.c). Anesthesia was induced with propofol (2 mg/kg, i.v.) and maintained with inhaled isoflorane (2%) in an oxygen carrier. Both rear limbs were clipped and subsequently scrubbed with 4% chlorhexidine gluconate solution for an aseptic preparation of the surgical field. With the animals in dorsal recumbency and after a small skin incision, the medial aspect of the femoral condyle was exposed by a medial skin, subcutaneous tissue and joint capsule incision (Fig. S1a). Using a 3.5 mm drill bit and under copious physiological saline irrigation to avoid thermal necrosis, a monocortical bone defect was performed trying to place it in the most center point of the femoral condyle (Fig. S1b). Afterwards, the defect was enlarged with a 5 mm drill bit under continuous physiological saline irrigation (Fig. S1c and d). The length of the defect was fixed at approximately 8 mm. The cylinders of the biomaterials were then softly press‐fitted in the bone defect (Fig. S1e). After assuring the good stability of the implants, the joint capsule, subcutaneous tissue, and the skin were routinely sutured in layers (Fig. S1f). The same procedure was repeated in the contralateral limb. In half of the animals, an additional implantation was performed by inserting a scaffold into the adjacent muscle tissue, also bilaterally. Once recovered from the anesthesia, the animals were housed again in the same individual boxes used for acclimatization and allowed to full weight bearing. During the postoperative period, analgesic (buprenorphine: 0.03 mg/kg s.c, b.i.d) and antibiotic (enrofloxcin: 10 mg/kg, b.i.d.) treatments were administered to the animals for 7 days. During the postoperative period, the animals were clinically assessed daily to ensure a healthy status with special focus on the detection of implant site infection, wound dehiscence, soft tissue swelling, discharge, loss of weight, etc. The animals with only osseous implants were euthanized at 4 weeks, and those with both osseous and intramuscular implants were euthanized at 12 weeks after the surgical procedure. Euthanasia was performed with an overdose of sodium pentobarbital (160 mg/kg i.v.) according to the legislation of the American Veterinary Medical Association (AVMA). A pre‐euthanasia sedation of midazolam (0.50 mg/kg s.c.) and medetomidine (0.05 mg/kg s.c.) was used for animal welfare reasons. For sample harvesting and histological procedure, the distal femoral regions and the intramuscular implants were explanted and fixed in 10 % neutral-buffered formalin solution for 24 h and immersed in 70 % ethanol solutions until further processing.

#### 2.8.3 Histomorphometric analysis by microCT

Quantification of bone volume in the intraosseous samples was performed by X-ray micro-computed tomography (MicroCT, SkyScan 1172, Bruker, MA, USA). MicroCT was performed at an accelerating voltage of 90 kV and a current of 111 μA with a Cu-Al filter. Images were acquired with a 10.4 μm pixel size. The exposure time and step resolution around the 360° were set to 2560 ms and 0.4° per step. An averaging of 3 frames per projection was performed. Reconstruction of cross sections was performed using software package NRecon (Bruker), which were subsequently aligned to the printing axis (DataViewer software, Bruker) and cylindrical volumes of interest (VOIs) corresponding to the scaffold region were selected using CTAn (Bruker).

Dragonfly software (version 2022.2.0.1409 for Windows, Comet Technologies Canada Inc., Canada, software available at http://www.theobjects.com/dragonfly) was used to measure the regenerated bone volume in the available volume (BV/AV), being the available volume the total volume minus the volume occupied by the scaffold.

#### 2.8.4 Histological processing

After microCT analysis, samples were divided in two halves along the longitudinal axis of the implant for the intraosseous implants. Intramuscular samples, on the other hand, were divided along the transversal axis (EXAKT 300 CP, Exakt Technologies, Germany). The first halves were dehydrated in an increasing series of ethanol solutions and embedded in increasing concentrations (i.e., 50, 70 and 100%) of methyl methacrylate (MMA) resin (Technovit 7200 VLC, Germany). Subsequently, the samples were stored overnight under vacuum and photopolymerised in a UV lamp (EXAKT 520, Exakt Technologies).

The second halves were decalcified in 10% ethylenediamine tetraacetic acid (EDTA) in PBS. Afterwards, samples were embedded in paraffin and cut using a microtome before performing histological stainings.

#### 2.8.5 Processing of undecalcified samples

The MMA resin blocks containing the undecalcified samples were polished down (Surface grinder, EXAKT 400 CS, AW110, Exakt Technologies) up to a P4000 grain size, coated with a carbon evaporated layer and analyzed by backscattered SEM (Phenom XL, Phenom World, MA, USA) operating at 15 kV for map imaging.

Afterwards, samples implanted in osseous defects were repolished to remove the carbon coating and Raman spectroscopy of selected groups was performed using a confocal Raman microscope (inVia™ Qontor® confocal Raman microscope, Renishaw Inc.). A 785 nm laser source with 1200 L/mm (vis) grating, 150 mW laser power, 0.1 s exposure time, and 30 accumulations were used for the measurement using a 50L × microscope objective lens. For each group, 3 samples were analyzed, taking 3 measurements from an area of regenerated bone and 3 from the native trabecular bone of the femoral condyle. The WiRE 4.4 software was used for all data collection. Data processing included a baseline correction and a 9-point smoothing.

One sample from each group of the 12-week timepoint implanted in osseous defects was selected to perform nanoindentation. Nanoindenter XP (MTS, MN, USA) equipped with the continuous stiffness measurement (CSM) module using a diamond, Berkovich indenter was used to measure hardness (H) and elastic modulus (E) from the regenerated bone as well as from the native trabecular bone of the femoral condyle. 25 nanoindentations were performed per material type with a maximum indentation penetration depth of ∼500 nm. The tip was adjusted using a fused silica standard, and the Poisson ratio was set at 0.3, which is representative of bone [54].

#### 2.8.6 Histological staining of decalcified samples

To assess general tissue morphology and bone quantification, hematoxylin and eosin (H&E) staining was performed in paraffin-embedded samples. For the detection of osteoclasts, staining for tartrate-resistant acid phosphatase (TRAP) activity was performed. QuPath v0.4.1 [55] was used to identify and quantify TRAP-positive cells and scaffold volume in the TRAP staining in at least two sections from each sample. Finally, a Masson trichrome (MT) staining was performed to assess tissue maturity.

### 2.9 Statistical analysis

Statistical analysis was performed using Graphpad Prism 8.0.1 (GraphPad Software Inc., San Diego, CA). Shapiro-Wilk test was used to assess normality. Significant differences between groups were determined using analysis of variance with Kruskal-Wallis multiple comparison tests. Results were considered significant at p values lower than 0.05 (p < 0.05).

## 3 Results and discussion

### 3.1 Characterization of the ion doping

The two proposed strategies allowed the fabrication of ion-doped CDHA scaffolds, each with different advantages and limitations. In all cases, the hardening of the scaffolds is based on the phase transformation from α-TCP to CDHA through the following reaction (Equation (1)):

Eq. 1 3Ca_3_(PO_4_)_2_ + H_2_O → Ca_9_(PO_4_)_5_(HPO_4_)OH

where the doping cations (Sr^2+^, Mg^2+^, Ga^3+^) and anions ((SiO_4_)^4-^) are expected to replace Ca^2+^ or the phosphate groups of the CDHA lattice, respectively, or to be incorporated in the hydrated layer of the CDHA nanocrystals.

The design of the strontium-doped ink was formulated in such a way that it did not require to modify the printing conditions, nor to incorporate other additives in the composition apart from the strontium salt, in order to be able to compare the biological response directly with the other groups. This limited the amount of strontium chloride incorporation to 20 wt% with respect to the powder phase. For the hardening process, it was necessary to avoid immersion in water, to prevent the leakage of the doping ion in the solution. In the second approach, the ions were incorporated into the soaking solution. To prevent scaffold disintegration upon immersion, a pre-setting step in 100 % relative humidity was performed. The duration of this step was deliberately minimized (3 h) to maximize the extent of the hydrolysis reaction leading to CDHA formation in the presence of the incorporated ions.

**Fig. 2:**
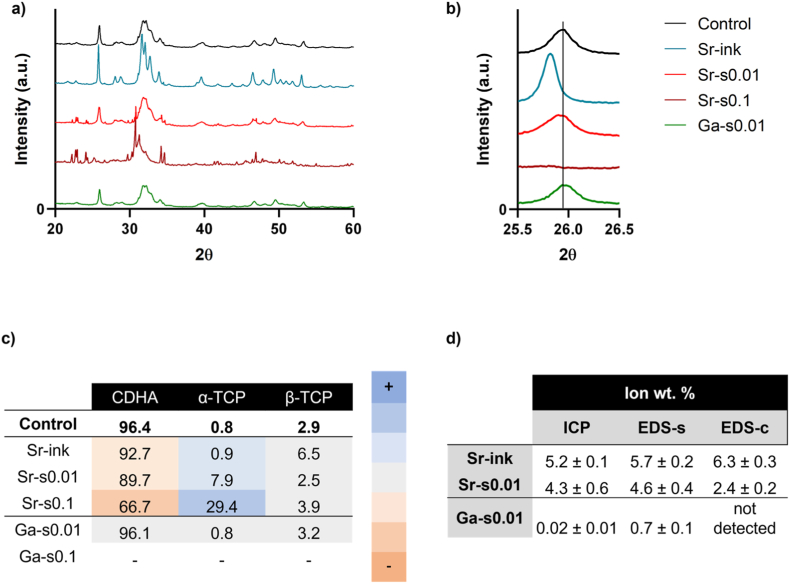
Chemical characterization of the Sr^2+^ and Ga^3+^ doped scaffolds. a) XRD diffractograms; b) magnification of the (002) peak; c) phase quantification by Rietveld refinement. Color code represents a value higher (in darker blue) or lower (in darker orange) than the control samples; and d) Ion quantification measured by ICP and EDS. For EDS, measurements were taken at the surface (EDS-s) and center (EDS-c) of the strand. (For interpretation of the references to color in this figure legend, the reader is referred to the Web version of this article.)

The powder XRD diffractograms of the doped scaffolds and the quantification of the crystalline phases are displayed in Fig. 2a–c for Sr^2+^ and Ga^3+^ and Fig. S2 for Mg^2+^and SiO_4_^4−^. As expected, the control group fully hydrolyzed to CDHA after 7 days (CDHA content 96.36 %). The incorporation of ions affected the reaction in different ways. The addition of Sr^2+^ in the ink did not hinder the conversion α-TCP to CDHA, resulting in scaffolds with a very similar hydrolysis rate. However, the resulting CDHA had a higher crystallinity. Although in stoichiometric HA, Sr^2+^ substitution generally decreases crystallinity due to lattice distortion arising from the ionic size mismatch (1.13 Å for Sr^2+^ vs. 0.99 Å for Ca^2+^), the situation with CDHA was different. It has to be considered that the CDHA lattice is intrinsically destabilized by Ca vacancies and associated distortions. At low substitution levels, Sr^2+^ incorporation can partially alleviate this instability by occupying vacancy-rich sites, thereby enhancing crystallinity relative to CDHA.

Surprisingly, unlike in the Sr-ink group, when Sr^2+^ was incorporated in the soaking solution, the transformation reaction was partially hindered, in a dose-dependent manner. Thus, immersion in the low-concentration soaking solution (Sr-s0.01) slightly delayed the phase transformation, but still, *ca.* 90 % of CDHA content was achieved after 7 days, whereas higher concentrations of Sr in the solution (i.e., Sr-s0.1) resulted in a smaller rate of transformation, with *ca.* 67% CDHA. This is in agreement with previous results from Boanini et al., who showed that the presence of Sr^2+^ in the soaking solution delayed the conversion of α-TCP to CDHA [56]. The same trend, but more marked, was found for the incorporation of Mg, which also hampered the hydrolysis reaction, achieving only *ca.* 58 and 5 wt% of CDHA for 0.01 and 0.1 mM Mg-containing solutions, respectively (Fig. S2a). This can be associated with the well-known inhibitory effect of Mg on apatite nucleation and crystallization [57]. Thus, the groups Sr-s0.1, Mg-s0.01 and Mg-s0.1 were discarded from further analysis. On the other hand, the incorporation of Si (Fig. S2b) and Ga (Fig. 2a–c) in the soaking solution did not interfere with the hydrolysis reaction to CDHA. However, the group Ga-s0.1 showed a loss of integrity during hardening and, therefore, was discarded from future analyses.

Ionic substitutions in a crystal induce lattice distortions, reflected as shifts in the diffraction peaks. Given that the ionic radius of Sr is larger than that of Ca, lattice expansion is anticipated, which should be reflected as a shift of the peaks toward lower angles, as previously reported [58]. In the case of Sr-ink scaffold, this shift was clearly observed in the (002) plane of CDHA (Fig. 2b), consistent with an enlargement of the unit cell dimensions in agreement with prior observations in Sr-Ca HA solid solutions [58,59]. By contrast, this shift was less pronounced in the Sr-s0.01 group. Two explanations may account for this observation. First, unlike the Sr-ink group, Sr^2+^ was introduced here via soaking, a process that involves diffusion from the surface into the scaffold. Consequently, Sr incorporation is expected to be higher at the surface than in the bulk of the material. Since powder XRD provides an averaged signal over the entire sample, this could explain the smaller overall shift. An alternative explanation is that, in this group, Sr was incorporated primarily into the hydrated layer rather than substituting Ca within the crystal lattice [15,16]. The ionic radius of Ga, on the other hand, is smaller than that of calcium so hence a contraction of the crystal lattice and a shift of the (002) peak towards larger values would be expected. Although very limited, a slight shift towards larger values suggested that the Ga was incorporated, at least to some extent, in the crystal lattice.

In order to assess the amount of doping ions incorporated in the scaffolds, the elemental composition was analyzed by ICP (Fig. 2d). The results revealed that the Sr-ink and Sr-0.01 groups contained 5.2 ± 0.1 and 4.3 ± 0.6 wt% of Sr, respectively. This is expected to have a biological effect as shown by other authors who have incorporated similar amounts [60,61]. In Ga-s0.01 scaffolds 0.02 wt% of Ga was incorporated. While this amount is small, Ga-doping has been previously studied from concentrations as low as 0.075 wt%, which is in the same range as our scaffolds [62]. On the other hand, Si-s0.01 and Si-s0.1 showed incorporations of 0.08 and 0.11 wt% of Si, respectively. Although these values are similar to those observed for Ga-s0.01, approximately 1 wt% of Si ions have been proposed to be necessary to positively influence bone growth, which is 10 times higher than what we achieved in our samples. Therefore, the amount of Si obtained here would not be expected to enhance bone regeneration [63], so these two groups were discarded from further analysis. It is worth noting that here we used TEOS as a source of Si, which has been commonly used in the literature [[63], [64], [65]], but alternative sources could be explored in future studies. For comparison, the ionic concentration, both on the surface and in the center of the cross-section of the 3D-printed filaments, was measured by Energy-dispersive X-ray spectroscopy (EDS) (Fig. 2d). The results highlight the effect of the different preparation methods on the distribution of the doping ions. For Sr-ink the Sr concentration at the center and on the surface of the 3D-printed strands were comparable amongst them and with the concentrations measured by ICP. In contrast, the Sr-s0.01 and Ga-s0.01 samples, where the ions were incorporated through the soaking solution, exhibited higher values on the surface than in the inner part, as expected due to the diffusion process. In the case of Ga, it was undetectable in the central part of the filaments. In the two materials the concentration on the surface was higher than the average value obtained by ICP.

#### 3.1.1 Characterization of the Sr^2+^- and Ga^3+^- doped CDHA scaffolds

After the chemical characterization by XRD and ICP, three experimental groups were selected, together with the control group, to perform a more in-depth characterization in terms of surface properties, porosity, mechanical properties, and ion release.

**Fig. 3:**
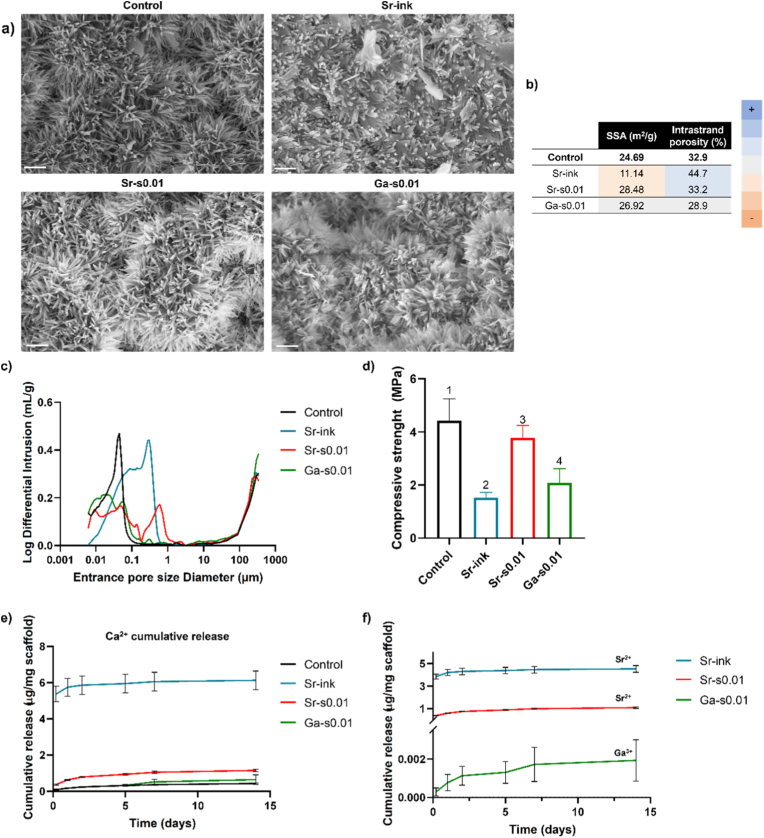
Material characterization of selected groups. a) SEM images of the microstructure of Control, Sr-ink, Sr-s0.01 and Ga-s0.01 samples; b) specific surface area (SSA) and intrastrand porosity measured by mercury intrusion porosimetry (MIP). Color code represents a value higher (in darker blue) or lower (in darker orange) than the control samples; c) Pore entrance size distribution obtained by MIP; d) Compressive strength (in MPa) of the printed scaffolds. Groups labelled with different numbers are statistically different (P < 0.05); e) cumulative release of Ca^2+^ and f) cumulative release of the incorporated ion (Sr^2+^ or Ga^3+^), normalized by mg of sample. (For interpretation of the references to color in this figure legend, the reader is referred to the Web version of this article.)

The SEM images (Fig. 3a) revealed the typical microstructure of CDHA for the control and Sr-s0.01 groups, consisting of an entangled network of needle-like nanocrystals, as shown in previous studies [66,67], resulting in similar SSA values (Fig. 3b). In contrast, the Ga-s0.01 group exhibited a slightly different crystal morphology, tending toward a plate-like structure with a lower aspect ratio. The Sr-ink group displayed shorter and thicker needles along with some plate-like crystals, which contributed to a lower SSA. It is known that the microstructure of CDHA is affected by the setting conditions [68]. Specifically, while most groups were immersed for 7 days in an aqueous solution, Sr-ink was kept for 7 days in 100% relative humidity to prevent leaching of the dopant ion. The absence of a liquid environment is expected to hinder ionic diffusion, which could explain the formation of shorter needles and plate-like nanocrystals [7]. Furthermore, the observed change in crystal morphology in Sr-ink but not in Sr-s0.01 is consistent with the higher incorporation of Sr into the crystal lattice in the former, as indicated by the XRD peak shift, which may lead to morphological changes as reported in previous studies [56].

On the other hand, a higher intrastrand porosity was registered by MIP in the Sr-ink group, which can be related to the high solubility of strontium chloride that was incorporated as a 20 wt% of the powder phase. As the LPR was not reduced to maintain the printability, the dissolution of the strontium chloride resulted in higher porosity and a larger pore entrance diameter (Fig. 3b–c) [69,70].

This higher porosity and smaller SSA in the Sr-ink group likely account for pronounced decrease in mechanical properties, as evidenced in Fig. 3d, where a marked reduction in compressive strength is shown. In contrast, no significant changes in porosity were detected for the other groups. For Sr-s0.01, the slight decrease in compressive strength may be attributed to incomplete α-TCP hydrolysis, whereas in the case of Ga-s0.01, where hydrolysis was complete, the reduction is most plausibly linked to the alterations in crystal morphology.

The evolution of the ion release (Ca^2+^, Sr^2+^ and Ga^3+^) in TRIS buffer, measured over 14 days, is shown in Fig. 3e and f. As expected, Sr-ink exhibited the highest Ca^2+^ and Sr^2+^ release, characterized by a significant initial burst followed by a sustained release over time. Sr-s0.01 scaffolds showed a similar pattern, although the total amount of released ions was notably lower. The initial burst release can be associated to the ions contained in the hydrated layer, which are more readily exchanged with the surrounding liquid. Moreover, the faster Ca^2+^ release from the Sr-containing scaffolds compared to the control can be associated to the higher solubility of Sr-doped HA, as reported in the literature [58,71,72]. The difference observed between the two Sr-containing groups could be attributed to the different ion incorporation and hardening methods. While Sr-s0.01 was soaked in an ion-containing solution during the hardening step and then rinsed before characterization, the hardening of the Sr-ink group was performed in a 100 % relative humidity environment to avoid leakage of the ions. Therefore, in the Sr-ink group some Sr^2+^ ions might remain physically entrapped in the crystal network and hence, they could be rapidly released when soaked. Similarly, all the Ca^2+^ ions that were replaced by Sr^2+^ ions in the lattice could also be immediately released when soaked. The higher porosity observed in the Sr-ink group could further facilitate ionic exchange with the surrounding liquid. Nevertheless, when comparing both groups, apart from the initial burst release, the release kinetics was very similar and higher than in the control group. As for the Ga^3+^ dopped scaffolds, the release of Ca^2+^ was very similar to the control, while there was a small and sustained release of Ga^3+^, consistent with the very low amount of Ga incorporated in this group.

### 3.2 Cytotoxicity assessment

The cytocompatibility of the 3D-printed scaffolds was evaluated via an indirect cytotoxicity extract assay over a 72 h period. As shown in Fig. S3, the metabolic activity of the cells remained high across most conditions, confirming the overall safety of the materials. While the undiluted (100%) extract from the Ga-s0.01 group exhibited a statistically significant reduction in cell viability compared to the medium-only control (p < 0.001), the viability remained above 50%, and this effect was completely neutralized upon dilution. All other experimental groups, including the strontium-doped inks and scaffolds (Sr-ink and Sr-s0.01), demonstrated excellent cytocompatibility across all dilution ratios, with viability values consistently comparable to or exceeding the control. These results suggest that while the concentrated gallium release may have a transient effect on cell metabolism in a closed *in vitro* system, the scaffolds are non-cytotoxic under more physiologically relevant, diluted conditions.

### *3.3* *In vivo* characterization

The *in vivo* assessment was performed using a rabbit femoral condyle model for analysis of the osteoconduction properties at 4 and 12 weeks and a rabbit intramuscular pocket model for analysis of the osteoinductive properties at 12 weeks.

Throughout the postoperative period, two animals of the 12-week group died from causes unrelated to the study and the samples were not included in the final assessment. This resulted in the loss of four samples, one from each group. The rest of the animals completed a normal postoperative period without any clinical complications. No signs of infection, wound dehiscence, weight loss, behavior changes, implant migration or lameness were observed during the postoperative period.

#### 3.3.1 Osteoconduction assessment

**Fig. 4:**
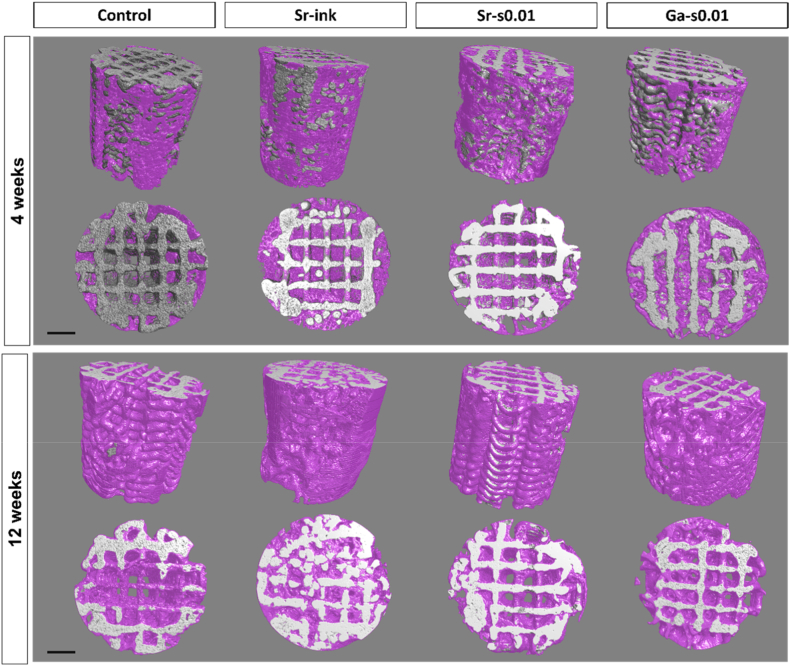
Bone segmentation by Dragonfly of 3D reconstructions from microCT data. Orthogonal and top views of a representative scaffold from each group after 4 and 12 weeks of implantation. Grey/white colors represent the scaffold and purple represents the regenerated bone. Scale represents 1 mm. (For interpretation of the references to color in this figure legend, the reader is referred to the Web version of this article.)

**Fig. 5:**
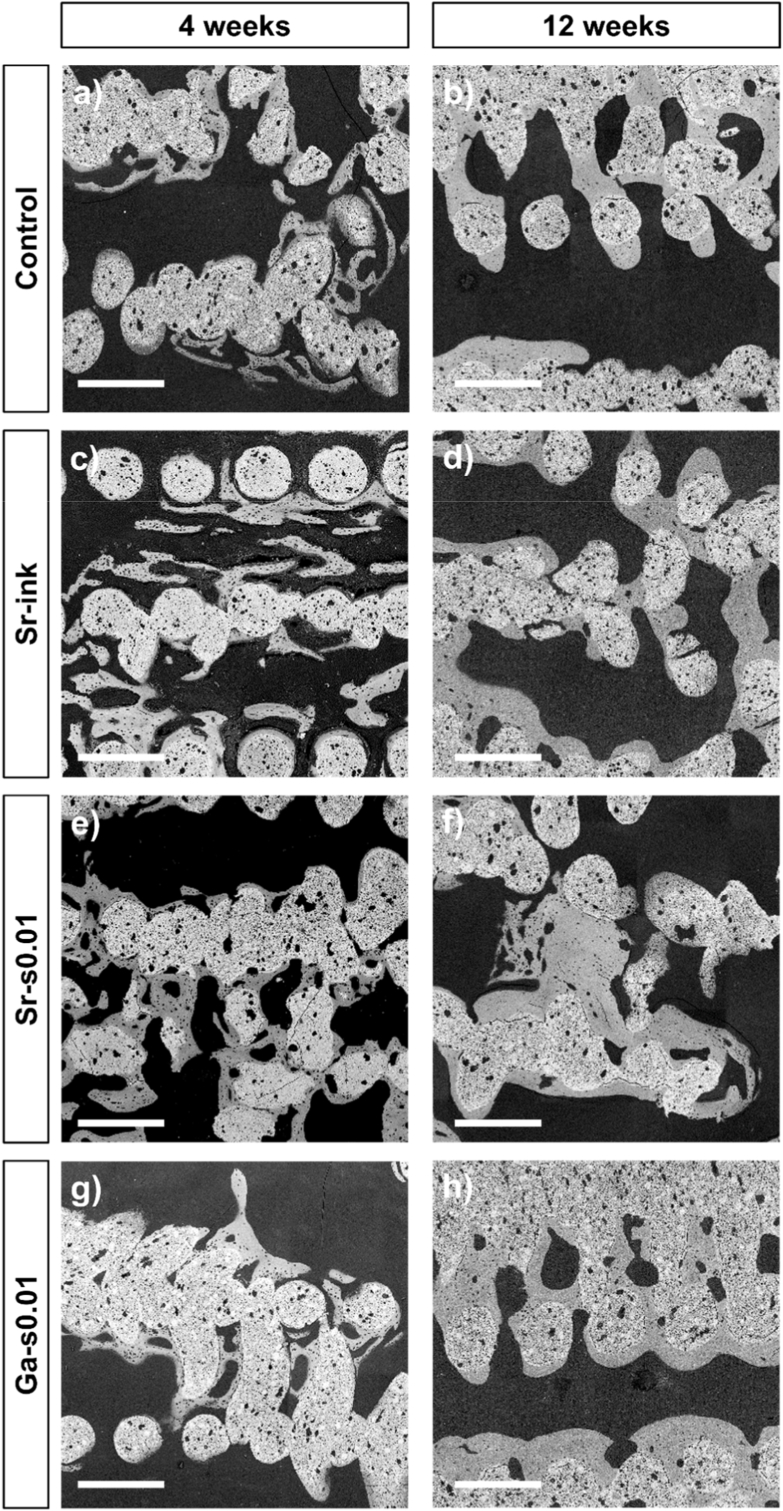
SEM images of representative samples of a) Control; b) Sr-s0.01; c) Sr-ink; and d) Ga-s0.01 after 12 weeks of implantation. Scale bar represents 500 μm.

**Fig. 6:**
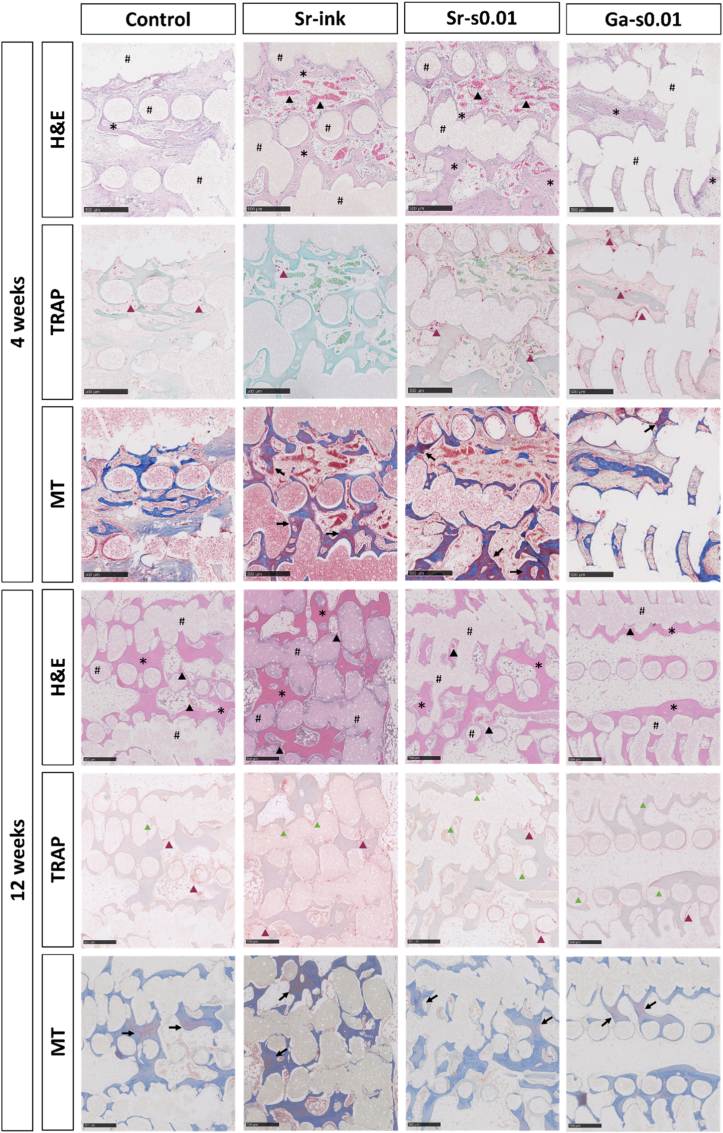
Decalcified histological sections of samples implanted in osseous defects during 4 or 12 weeks. Representative micrographs of consecutive sections in each group stained by H&E, TRAP and MT stainings. In H&E staining, * indicates new bone formation, # indicates remaining scaffold and black arrowheads indicate blood vessels. These elements can be observed in all three stainings but are only indicated in the H&E micrographs for clarity. In TRAP staining, red arrowheads indicate TRAP-positive cells and green arrowheads (only in the 12 week sections) indicate scaffold degradation. Scale bar represents 500 μm. (For interpretation of the references to color in this figure legend, the reader is referred to the Web version of this article.)

After retrieval of the samples, microCT (Fig. 4), SEM (Fig. 5) and histologies (Fig. 6) were performed to analyze bone formation qualitatively and quantitatively. Reconstruction of the microCT images (Fig. 4) revealed that after 4 weeks of implantation, some bone had already regenerated in all groups, but the amount of bone in the center of the scaffold was small, especially in the control and Sr-s0.01 groups. Sr-ink and Ga-s0.01, on the contrary, showed a higher bone infiltration. After 12 weeks, full coverage of the outer part of the scaffolds and higher infiltration towards the center was observed in all groups.

In agreement with the microCT results, the SEM micrographs performed on undecalcified samples showed newly formed bone growing in the form of thin trabeculae attached to the scaffold mainly in small porosity areas, except for Sr-ink where bone was also observed in larger pores (Fig. 5). After 12 weeks, most of the struts of the scaffolds were covered in bone and thick trabeculae connected strands across large porosity areas.

Three histological stainings were performed on decalcified samples; H&E, TRAP and MT (Fig. 6 and Fig. S4 at lower magnification). Similarly to what was observed in SEM, H&E staining showed thin trabeculae covering the strands of the scaffolds after 4 weeks of implantation, which became thicker and better interconnected after 12 weeks. In both cases, newly-formed bone consisted on a mixture of woven and lamellar bone, and some Harversian channels were observed. Furthermore, H&E staining showed no fibrous capsule at the bone-material interface. A light inflammatory reaction was observed, mainly in the bone marrow at both time points in some samples of all groups. Additionally, minimal fibrous tissue infiltration was observed in some samples. All samples were well vascularized, some with only small capillaries, but most of them showing large blood vessels.

TRAP staining was conducted to assess the presence of osteoclasts. TRAP-positive cells were observed at both time points, always at the scaffold or bone surfaces. While no degradation was visible at week 4, some resorption pits were observed after 12 weeks, with bone ingrowth into the created porosity.

Finally, MT staining provided complementary information, as it showed the presence of well-structured collagen, stained in blue, in all samples [73].

**Fig. 7:**
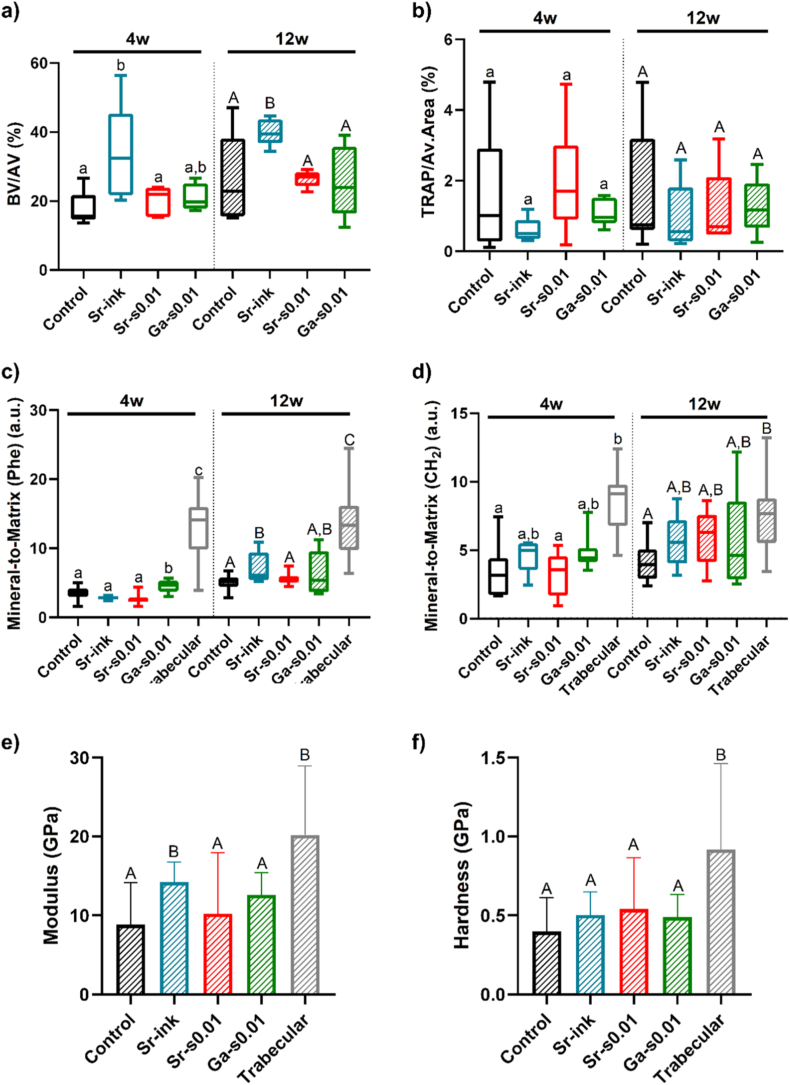
Characterization of the newly formed bone. a) Histomorphometrical results: percentage of newly formed bone volume within the available volume (BV/AV) at 4 and 12 weeks post-implantation, as measured by microCT (n = 5); b) Percentage of TRAP-positive areas with respect to the available area (TRAP/Av. Area); c-d) Mineral-to-matrix ratio of the host trabecular bone and the regenerated bone measured by Raman spectroscopy, taking into account different characteristic bands of the matrix, either phenylalanine (c) or the proteins with CH2 deformation (d); e) Young's modulus and f) hardness of the newly formed bone in the different groups compared to the host trabecular bone, as measured by nanoindentation after 12 weeks of implantation. Different letters denote groups with statistically significant differences at the same time point (p < 0.05). Lower case letters indicate differences within the 4-week time point, while capital letters indicate differences within the 12-week time point.

The characterization of the newly formed bone is shown in Fig. 7. Quantification of bone volume and TRAP-positive cells was performed using the microCT reconstructions and TRAP staining respectively, while bone quality was assessed via Raman microspectroscopy and nanoindentation. The Sr-ink group exhibited significantly greater bone formation than the control at both time points, whereas no statistically significant differences were detected for the Sr-s0.01 and Ga-s0.01 groups (Fig. 7a). The markedly improved biological performance of Sr-ink compared to Sr-s0.01 may be attributed to the higher Sr^2+^ content and increased porosity, both of which promoting greater ion release. This enhanced ion release likely triggered an earlier biological response and stimulated bone formation. In addition to the effects associated to Sr^2+^ release, the higher intrastrand porosity of Sr-ink scaffolds may have directly supported bone formation, consistent with previous reports that microporosity enhances osteogenesis [52,74,75].

In contrast, Ga-s0.01, did not promote bone formation relative to the control. Although Ga^3+^ doping of calcium phosphates has been previously investigated at concentrations as low as 0.075 wt%, which is in a similar range to our scaffolds, *in vivo* osteogenic responses have only been demonstrated at higher levels (0.3 wt%) [62,76]. Therefore, the absence of a positive effect in this study is likely attributable to the low Ga^3+^ content incorporated in Ga-s0.01 scaffolds.

Surprisingly, no statistically significant differences were found between the 4- and the 12-week time points. However, qualitative analysis of SEM micrographs and histological sections revealed that bone regenerated bone after 12 weeks exhibited thicker, more continuous trabeculae compared to that observed at 4 weeks post implantation. Moreover, as shown in Fig. S5, bone-scaffold segmentation performed in Dragonfly may overestimate the amount of bone at 4 weeks, due to the presence of thin trabeculae, whereas segmentation accuracy is improved at 12 weeks, reflecting the maturation and consolidation of the newly formed bone. It is also important to note that the high variability observed in bone formation is inherent to the biological complexity of the *in vivo* model. The dispersion typically stems from individual physiological differences, such as metabolic rates and endogenous hormonal profiles, among the animal subjects. Furthermore, small variations in the initial contact between the scaffold and the surrounding host tissue (the “bone-implant interface”) can significantly influence the rate of cellular infiltration and vascularization. These localized micro-environmental differences, can significantly influence the pace of bone formation. This high variability has been previously observed in other studies using similar biomaterial scaffolds [77,78].

Contrary to expectations, given that both strontium and gallium are known to inhibit osteoclastogenesis [29,47], TRAP quantification revealed no statistically significant differences between groups, with osteoclast activity levels remaining similar across all groups throughout the study. To further contextualize these findings, we compared our measured ionic release with established therapeutic thresholds. While Ga^3+^ concentrations of 50-200 μM are typically required to suppress osteoclast activity via proton-pump inhibition [79,80], the trace release observed in our Ga-s0.01group (0.002 μg/mg) is significantly lower. Similarly, while Sr^2+^ is known to inhibit osteoclastogenesis at concentrations between 0.1 and 1 mM [29], the 5 μg/mg released by the Sr-ink scaffolds in a high-turnover rabbit model was likely insufficient to reach the sustained local molarity necessary to alter TRAP-positive populations.

Bone quality was next evaluated in comparison with native trabecular bone using Raman spectroscopy and nanoindentation (Fig. 7c–f). As recently demonstrated by, Lodoso-Torrecilla et al. [77], Raman spectroscopy provides a reliable means to monitor bone regeneration quality in scaffold-based strategies. Specifically, this technique enables the determination of the mineral-to-matrix ratio, with the phosphate band (ν_1_PO_4_^3^) at 959 cm^−1^ serving as the indicator of the mineral phase [81], and the phenylalanine (with a band at 1003 cm^−1^) [82] and the CH_2_ deformation band present in the collagen (at 1450 cm^−1^) as indicators of the organic phase of bone [83]. After 4 weeks, all groups exhibited significantly lower mineral-to-matrix ratios than native trabecular bone. However, Ga-s0.01 and, to a lesser extent, Sr-ink showed comparatively higher ratios. After 12 weeks, the mineral-to-matrix ratios increased and approached those of native trabecular bone. Interestingly, when analysis was restricted to the CH_2_ deformation band, all ion-doped groups had mineral-to-matrix ratios comparable to trabecular bone (p > 0.05), whereas the non-doped group remained significantly lower (p < 0.05). Overall, these results indicate that bone regenerated in Ga-s0.01and Sr-ink scaffolds exhibited higher mineralization than the control. In the case of Sr-ink, this coincided with a greater amount of bone, whereas Ga-s0.01 promoted improved mineral quality without increasing bone quantity. The improved bone quality observed in the gallium-containing scaffolds aligns with previous literature. Piñera-Avellaneda showed that a small release of gallium, in the order of 300-600 ppb, increased hMSCs mineralization [47]. More importantly, treatment with gallium nitrate has been shown to increase mineral content and bone density *in vivo* [[84], [85], [86]], which can be attributed to the physical-chemical integration of Ga^3+^ into the bone mineral phase. As previously demonstrated, gallium ions can be incorporated in the apatite crystals, altering their dissolution kinetics [87]. This suggests that the gallium released from the Ga-s0.01 scaffolds acts as a mineral stabilizer, reinforcing the structural integrity of the newly formed bone even in the absence of an overall increase in bone volume.

The mechanical properties of the regenerated bone were assessed by nanoindentation only after 12 weeks of implantation, as the thin trabeculae present at 4 weeks precluded reliable measurements. After 12 weeks, the Sr-ink group exhibited a significantly higher elastic modulus, comparable to that of native trabecular bone, whereas the other groups displayed lower values. Notably, a positive correlation was observed between the elastic modulus and the mineral-to-matrix ratio determined by Raman spectroscopy using the phenylalanine band. No statistically significant differences were detected between groups with respect of bone hardness.

#### 3.3.2 Osteoinduction assessment

Material-driven osteoinduction refers the capacity of a material to initiate bone formation by inducing the differentiation of undifferentiated cells into the osteogenic lineage. In this study, osteoinduction was evaluated in an ectopic model using intramuscular defects in rabbits over a 12-week period. This model presents inherent challenges due to the recognized species-dependent nature of the osteoinduction, which is more readily observed in large animals, such as dogs and pigs, and less so in rodents [88]. In rabbits, ectopic bone formation is particularly limited, and has been reported to occur at lower levels than in other rodents like mice [89]. Nonetheless, this model was selected in accordance with the ethical principles of the 3Rs (Replacement, Reduction and Refinement), enabling the simultaneous evaluation of both orthotopic and ectopic implantation sites and thereby reducing the overall number of animals required. Moreover, previous studies have shown that concave surface features strongly promote osteoinduction, whereas convex geometries, such as those predominant in the strands of the 3D-printed scaffolds studied here, are less favorable [4]. Given these constraints, the aim was to investigate whether the incorporation of ions could overcome these limitations. Ectopic bone formation was detected in two animals implanted with Sr-ink scaffolds and in one animal implanted with Ga-s0.01 scaffolds, while no osteoinductive response was observed in the control or Sr-s0.01 groups (Table 1).

**Table 1:** Bone incidence after 12 weeks of implantation in intramuscular defects.

Group	Incidence of bone formation
Control	0/6
Sr-ink	2/5
Sr-s0.01	0/5
Ga-s0.01	1/5

It has to be emphasized that the ectopic bone formation observed in our study was a result of a regulated, cell-mediated process, where the material surface properties and ionic release provided the necessary signals for mesenchymal stem cell recruitment and differentiation, as previously shown in other studies [4,88,89]. Crucially, this effect is localized to the scaffold microenvironment and does not imply a risk of systemic or uncontrolled soft-tissue calcification. In an orthotopic setting, this osteoinductive potential acts synergistically with the host endogenous healing signals, merely accelerating the natural regenerative timeline, as we previously demonstrated [3].

**Fig. 8:**
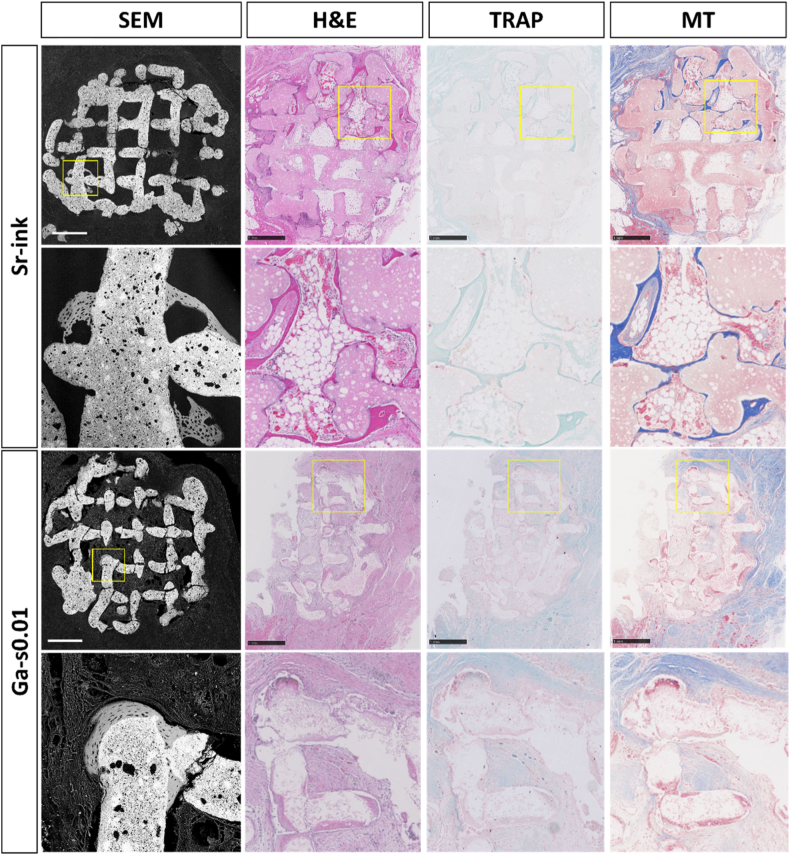
Histological findings at week 12 of implantation in intramuscular defects of the Sr-ink and Ga-s0.01 groups. Undecalcified samples were observed by SEM while decalcified samples were stained with H&E, TRAP and MT stainings. Scale bar represents 1 mm.

Fig. 8 presents SEM micrographs along with HE, TRAP and MT staining of Sr-ink and Ga-s0.01 specimens that exhibited osteoinductive properties. Bone was clearly visible in the SEM images of both groups. In the Sr-ink group, histological staining revealed also abundant bone formation, whereas no bone was detected in the Ga-s0.01 group likely reflecting the inherently sparse and localized nature of bone occurrence in ectopic models. Fig. S6, on the other hand, shows representative images of the control and Sr-s0.01 groups, which did not show osteoinduction.

Vascularization was assessed, and no clear correlation was observed between angiogenesis and ectopic bone formation, as samples within all groups displayed variable levels of neovascularization, ranging from abundant to minimal. TRAP staining indicated low osteoclastic activity overall, with only a few specimens showed TRAP-positive cells, which is consistent with the expected temporal progression of osteoinduction. Hayashi and Ishikawa investigated osteoinduction in rabbits using CaP scaffolds in intramuscular defects for 1, 2, 3, 4, 8, and 12 weeks [90]. They reported emergence of TRAP-positive cells between weeks 2-3, a decline by weeks 3-4, and subsequent osteoid and mineralized tissue formation at weeks 8 and 12, corresponding to what was observed in the present study [[91], [92], [93]].

Given the aforementioned challenges, the observation of osteoinduction in two experimental groups represents a significant achievement. Among them, Sr-ink exhibited the most promising results, demonstrating both the highest amount of bone and the greatest prevalence of ectopic bone formation. In contrast, Sr-s0.01 showed no osteoinductive properties. These differences are likely attributable to the higher ion release and the greater microporosity of Sr-ink scaffolds, as increased strut microporosity in CaP scaffolds has been linked to enhanced ectopic bone formation [89]. Notably, the osteoinductive response observed in the Ga-s0.01 group, despite its minimal Ga^3+^ content, highlights the ion’s potential and supports further investigation at higher concentrations. The known antimicrobial properties of Ga^3+^ [43,44,46] further underscores its promise as a multifunctional additive for synthetic bone grafts. To date, claims of Ga^3+^ associated osteoinduction have been based primarily on *in vitro* studies [47,94] or on *in vivo* orthotopic models [76]. The Ga^3+^ concentrations used here (400 ppb) were comparable to those reported by Piñera-Avellaneda [47] in *in vitro* studies (280–570 ppb, demonstrating dose-dependent effects), yet an order of magnitude lower than concentrations employed by Strazic Geljić et al. [76] in orthotopic *in vivo* models. To the best of our knowledge, although the incidence of ectopic bone formation was low, this study provides preliminary evidence that Ga^3+^ may mediate osteoinduction in a relevant ectopic *in vivo* model, suggesting its potential role in bone tissue engineering.

## 4 Conclusion

In this study, we proposed two straightforward approaches to incorporate ions into 3D printed CaP scaffolds. We successfully incorporated Sr^2+^ by direct addition into the ink formulation and Sr^2+^ and Ga^3+^ by soaking the printed scaffolds in ionic solutions. Post-printing incorporation by soaking in ionic solutions, while simpler, was less effective. Overall, Sr-ink had the most promising *in vivo* results, showing higher ion loading and release, with enhanced bone formation and bone quality compared to non-doped scaffolds and demonstrating its osteoinductive properties. Ga-s0.01 enhanced bone quality, and most importantly, its osteoinductive properties were demonstrated for the first time in an ectopic *in vivo* model. Further studies should focus on increasing the concentration of Ga to further improve its performance.

## CRediT authorship contribution statement

**Irene Lodoso-Torrecilla:** Data curation, Formal analysis, Investigation, Methodology, Writing – original draft. **Daniel Moreno:** Investigation, Writing – review & editing. **Gaël Ciucci:** Investigation, Writing – review & editing. **Miguel Mateu-Sanz:** Investigation, Methodology, Writing – review & editing. **Ji-Young Yoon:** Investigation, Writing – review & editing. **Emilio Jimenez-Pique:** Investigation, Writing – review & editing. **Jordi Franch:** Investigation, Writing – review & editing. **Maria-Cristina Manzanares:** Investigation, Writing – review & editing. **Joanna Konka:** Investigation, Writing – review & editing. **Montserrat Espanol:** Formal analysis, Investigation, Writing – review & editing. **Maria-Pau Ginebra:** Conceptualization, Formal analysis, Funding acquisition, Investigation, Supervision, Writing – review & editing.

## Declaration of competing interest

The authors declare that they have no known competing financial interests or personal relationships that could have appeared to influence the work reported in this paper.

## Data Availability

Data will be made available on request.
