# Supplementary material for: Strontium and gallium doping enhances in vivo bone regeneration in biomimetic hydroxyapatite 3D-printed scaffolds
Source: Mater Today Bio. 2026 Apr 16;38:103131. doi: 10.1016/j.mtbio.2026.103131 (PMC13101743; doi:10.1016/j.mtbio.2026.103131)
Supplement: Multimedia component 1 [file mmc1.docx]

**Strontium and gallium doping enhances *in vivo* bone regeneration in biomimetic hydroxyapatite 3D-printed scaffolds**

Irene Lodoso-Torrecilla^1,2^, Daniel Moreno^1,2^, Gaël Ciucci^1^, Miguel Mateu-Sanz^1,2,3^, Emilio Jimenez-Pique^2,4^, Jordi Franch^5^, Maria-Cristina Manzanares^6^, Joanna Konka^1,2^, Montserrat Espanol^1,2,3^, Maria-Pau Ginebra^1,2,3,7*^

^1^*Department of Materials Science and Engineering, Group of Biomaterials, Biomechanics and Tissue Engineering, Universitat Politècnica de Catalunya (UPC), Barcelona, Spain*

^2^*Barcelona Research Centre for Multiscale Science and Engineering, Universitat Politècnica de Catalunya (UPC), Barcelona, Spain.*

*^3^Centro de Investigación Biomédica en Red—Bioingeniería, Biomateriales y Nanomedicina (CIBER-BBN), Instituto de Salud Carlos III, Spain*

^4^ *Department of Materials Science and Engineering, CIEFMA Group, Universitat Politècnica de Catalunya (UPC), Barcelona, Spain*

^5^*Bone Healing Group, Small Animal Surgery Department, Veterinary School, Universitat Autonoma de Barcelona, 08193 Bellaterra, Barcelona, Spain*

*^6^Human Anatomy and Embryology Unit, Department of Pathology and Experimental Therapeutics, Universitat de Barcelona, 08907 L’Hospitalet de Llobregat (Barcelona), Spain*

^7^*Institute for Bioengineering of Catalonia (IBEC), Barcelona Institute of Science and Technology, Carrer Baldiri Reixac 10- 12, 08028 Barcelona, Spain*

*Corresponding author:

Maria-Pau Ginebra

Department of Materials Science and Engineering, Universitat Politècnica de Catalunya, Av. Eduard Maristany 16, 08019 Barcelona, Spain

Email: maria.pau.ginebra@upc.edu


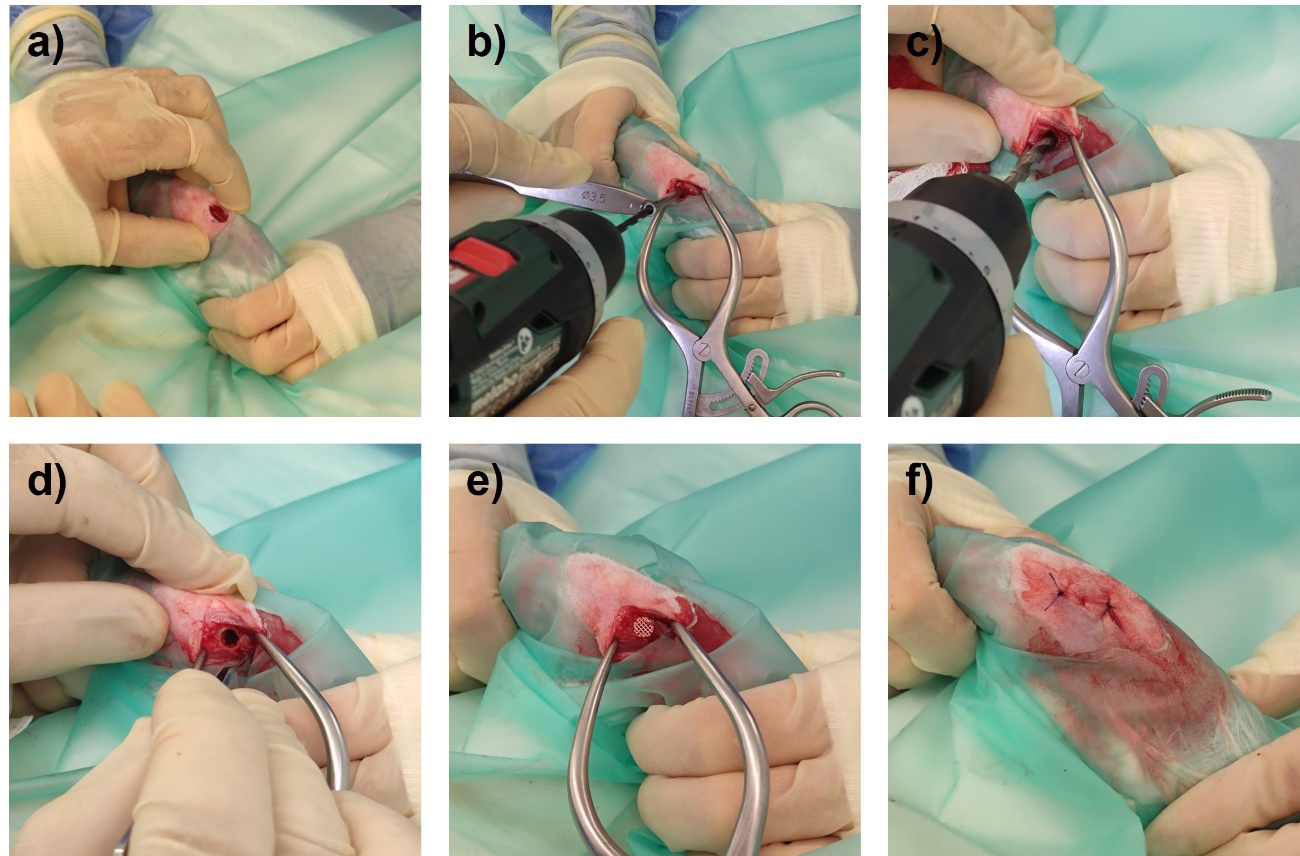


**Figure S1:** Overview of the surgical procedure: a) skin and subcutaneous incision; b) creating the defect in the medial femoral condyle with a Ø 3.5 mm drill; c) enlarging the defect with a Ø 5 mm drill (c); d) caudal view of the distal femur with the defect created and e) with the scaffold fully implanted. f) Surgical wound sutured.


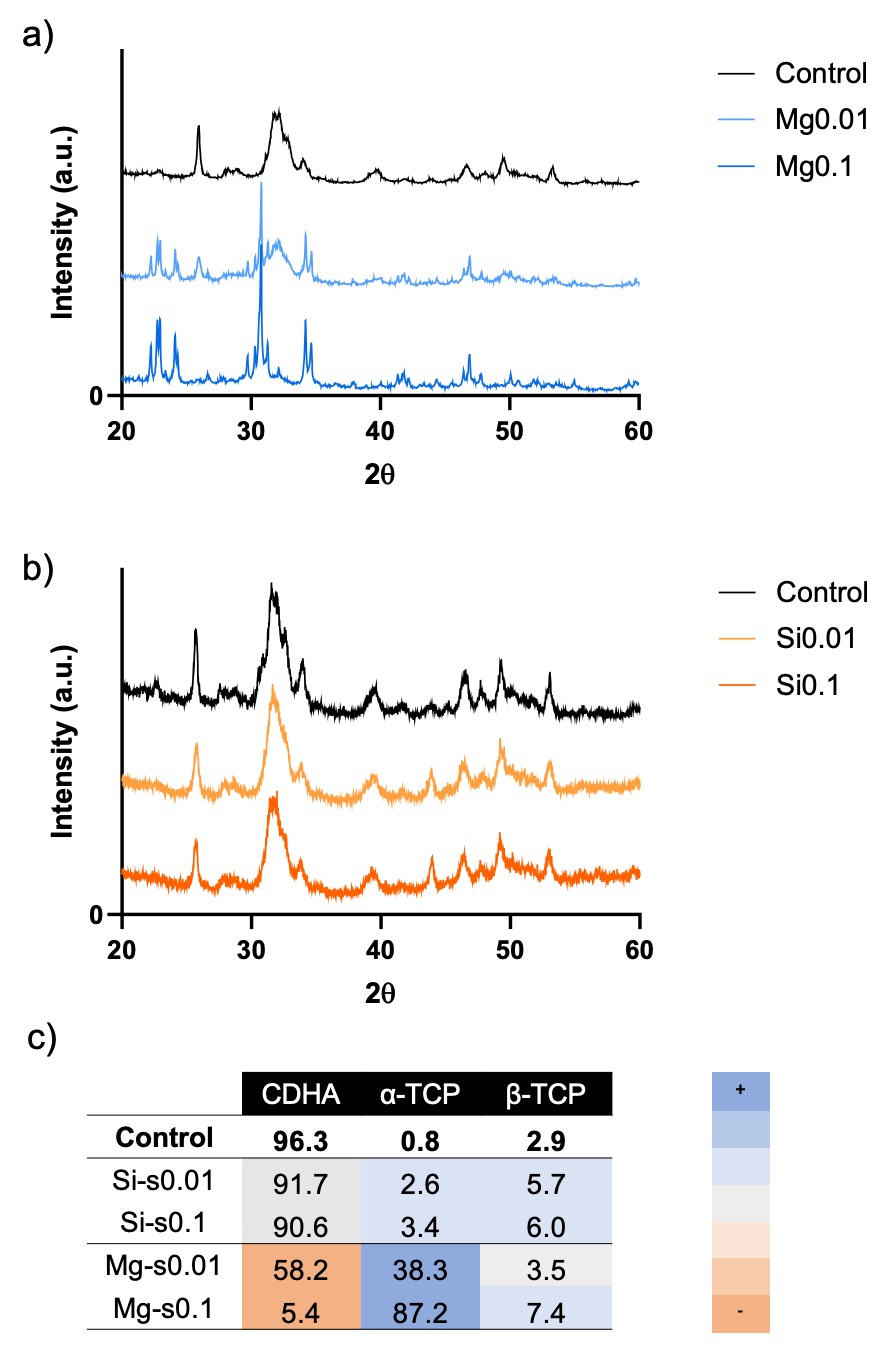


**Figure S2:** Chemical characterization of the excluded groups: XRD of a) Mg-containing groups and b) Si-containing groups and c) their phase quantification.

**Figure S3:** Cytotoxicity test of extracts from 3D scaffolds. The scaffolds were immersed in growth medium for 72 h, and the collected extracts were applied to Saos-2 cells. After 24 h, cell viability was assessed using a CCK-8 assay. The values were normalized to the corresponding medium-only group for each extract concentration. Differences between groups were evaluated using two-way ANOVA followed by Dunnett's multiple comparison test. Only statistically significant differences are indicated in the graph. **p* < 0.05, ***p* < 0.01, ****p* < 0.001, *****p* < 0.0001; n = 3 biological replicates. Results are reported as mean ± SD.


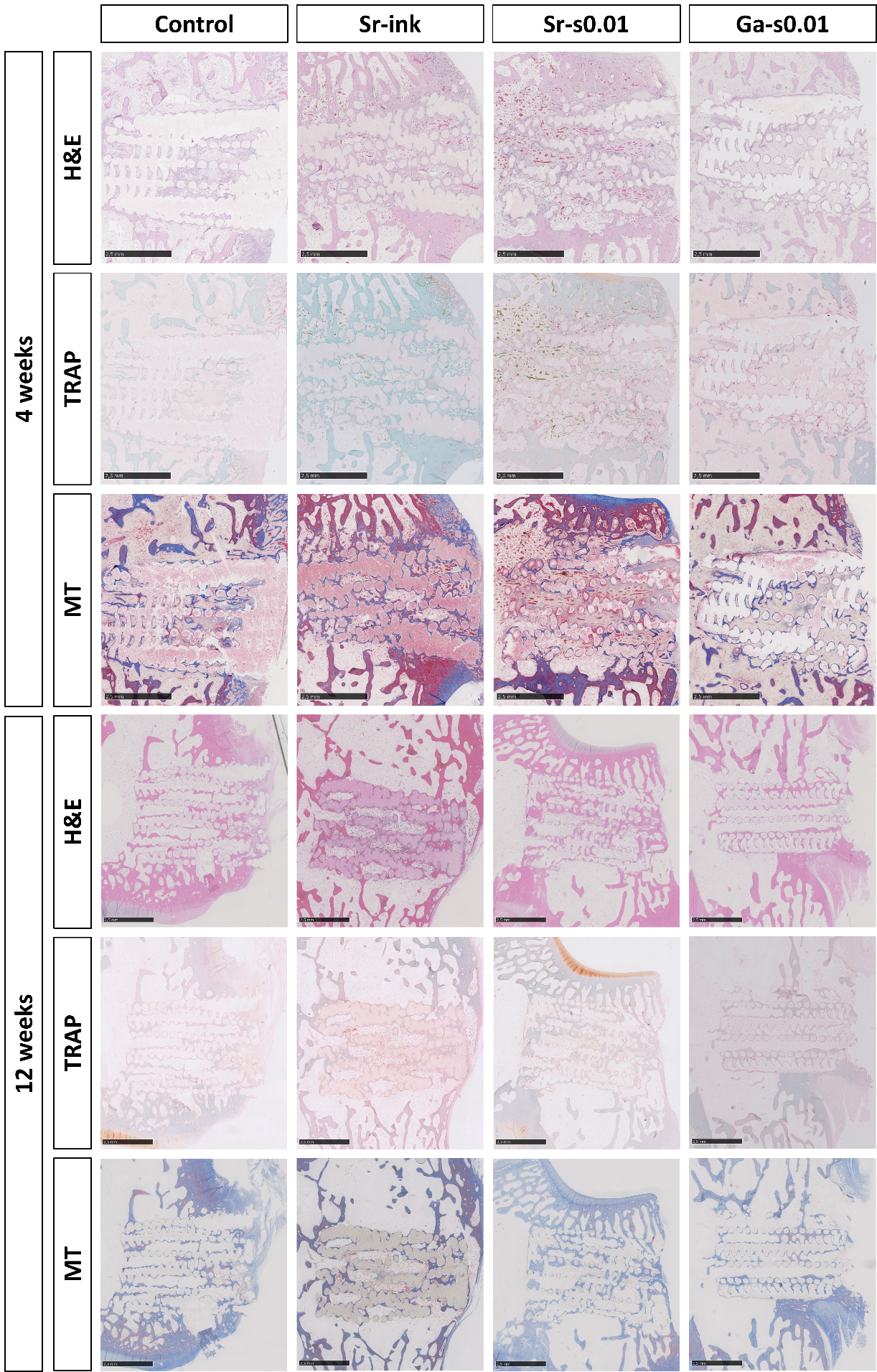


**Figure S4:** Decalcified histological sections of samples implanted in osseous defects during 4 or 12 weeks. Representative micrographs of consecutive sections in each group stained by H&E, TRAP and MT stainings. Scale bar represents 500 µm.

**Figure S5:** Illustration of the cross-section of a microCT sample implanted for a) 4 weeks or c) 12 weeks; and their segmentations performed by Dragonfly (b and d, respectively). In all images, white represents the scaffold, grey represents the bone and black represents the background. It can be observed that segmentation of the 4 weeks samples was less accurate, which could lead to some degree of overestimation.


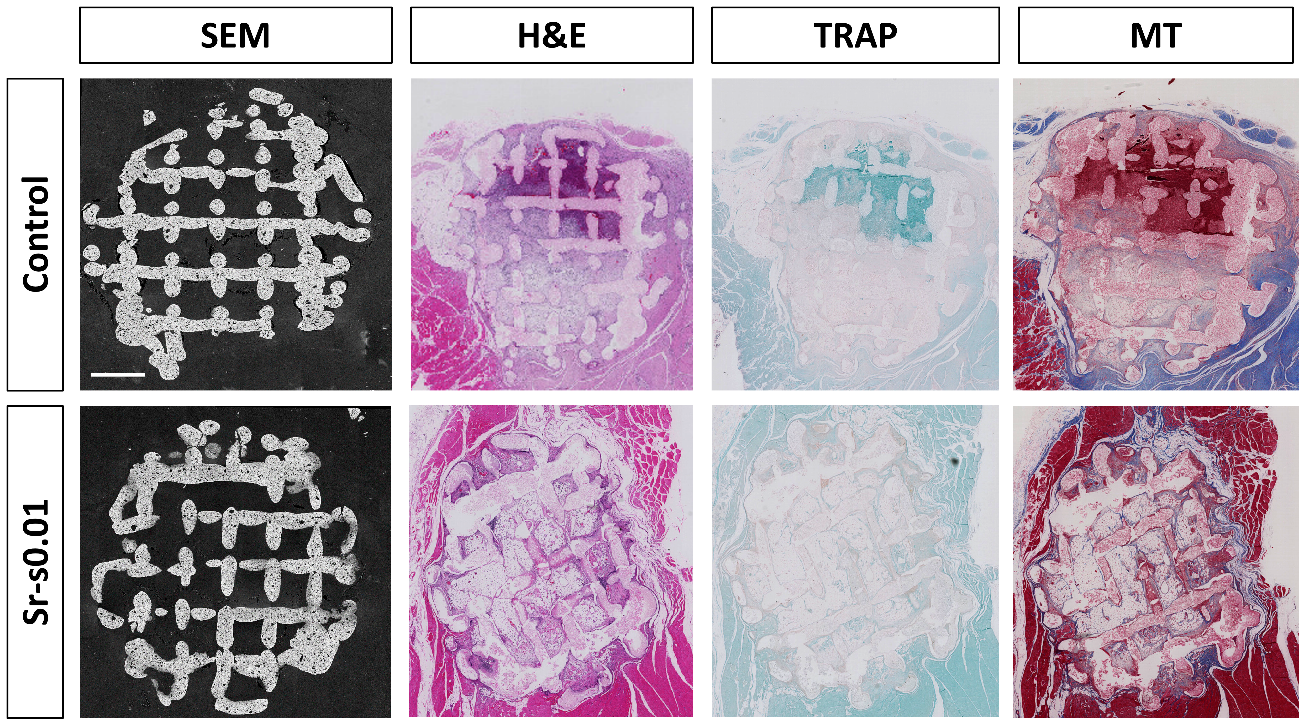


**Figure S6:** Histological findings at week 12 of implantation in intramuscular defects of the Control and Sr-s0.01 groups. Undecalcified samples were observed by SEM while decalcified samples were stained with H&E, TRAP and MT stainings. Scale bar represents 1mm.
